# Low-Energy Intraoperative Radiation Therapy and Competing Risks of Local Control and Normal Tissue Toxicity

**DOI:** 10.3389/fonc.2017.00212

**Published:** 2017-09-21

**Authors:** Geraldine M. Jacobson, Ramon Alfredo Siochi

**Affiliations:** ^1^Department of Radiation Oncology, WVU Cancer Institute, West Virginia University, Morgantown, WV, United States

**Keywords:** radiotherapy, breast cancer, breast conserving therapy, accelerated PBI, intraoperative radiation therapy, cardiac toxicity, lung toxicity

## Introduction

Radiotherapy (RT) following breast-conserving therapy has been demonstrated to reduce the risk of breast cancer local recurrence and death (1). The adverse normal tissue effects of radiation have been reduced, but not eliminated, with modern treatment planning techniques. Since the introduction of breast-conserving therapy in the 1980s, analyses of long-term results of randomized trials have identified patients that derive the most benefit in local control and disease-free survival. Partial breast irradiation (PBI) is an option for patients with defined, low-risk features that has the advantage of reduced radiation to the heart, lungs, and soft tissues. Low-energy intraoperative radiation therapy (IORT) is a subset of PBI that shortens the treatment course and reduces the dose to larger volumes of normal tissues compared to other techniques.

Patients with a lower risk of local recurrence are likely to live decades beyond their treatment and are at continued risk of impaired cardiac and pulmonary function. For these patients, it seems prudent to select treatments that reduce the risk of late radiation effects.

## Whole Breast Irradiation (WBI) and Local Control

While the proportional benefit of breast radiation in achieving local control is similar for all groups, the absolute benefit is related to patient and tumor characteristics. Data from the Early Breast Cancer Trialists’ Collaborative Group showed that the absolute benefit of post-BCS conserving RT was greater in younger women; 10-year risk reduction was 24.6% for women younger than 40 years of age and 8.9% for women 70 years and older (2). An update of the European Organization for Research and Treatment of Cancer Boost or No Boost trial showed a cumulative incidence of breast tumor recurrence (IBTR) at 20 years of 34% in patients younger than 40 years of age and 11% in patients 50 years and older (3). Young age and presence of ductal carcinoma *in situ* were the predominant factors associated with IBTR. Patients with high invasive grade were at high risk for IBTR in the first 5 years posttreatment, but this effect declined with time. Liu et al. showed that breast cancer subtype was related to IBTR in node-negative patients older than 50 years but not associated with response to radiation (4).

In 1985, the reported 5-year ipsilateral breast recurrence rate following lumpectomy and radiation from the National Surgical Adjuvant Breast and Bowel Project B06 trial was 7.7% (5). This level of local control supported the introduction of breast conservation as an acceptable treatment for early breast cancer. Local recurrence rates following conservative surgery and radiation are lower in modern series, probably related to more effective systemic therapy and improved surgical and pathology techniques.

## Accelerated PBI

For localized breast cancer, the most common site of recurrence is at or near the index lesion. Recurrence in other quadrants is not impacted by WBI (6). Accelerated PBI (APBI) treats a limited volume surrounding the tumor cavity with larger fractions over a shorter period than WBI. Advantages of APBI include reduced treatment time, fewer patient visits, and reduced normal tissue effects. Several treatment delivery methods are utilized, including interstitial, intracavitary, intensity-modulated radiation therapy or 3-D conformal, proton therapy, and IORT.

A systematic review of eight randomized trials of APBI showed a small difference of local recurrence in favor of WBI but no difference in nodal recurrence, systemic recurrence, overall survival, or mortality (7). A randomized, non-inferiority trial of multicatheter brachytherapy versus WBI reported local recurrence of 1.44% with APBI versus 0.92% with WBI at 5 years, demonstrating non-inferiority with respect to local control and disease-free and overall survival (8). Several studies have identified age as a risk factor for local recurrence with APBI, with lower risk in patients older than 50 years (9, 10). This is reflected in the American Society for Radiation Oncology consensus statement regarding APBI, with age of 50 years or older in the suitable category (11). APBI has been widely used for decades and tested in clinical trials in over 1,000 patients. For appropriately selected patients, outcomes are comparable to WBI. In addition, there is a lower incidence of non-breast cancer mortality. A meta-analysis of randomized trials of PBI versus whole breast radiation reported lower 5-year non-breast cancer and overall mortality rates in patients treated with PBI, amounting to a 25% reduction in relative terms (12).

## Low-Energy X-Ray IORT

Intraoperative radiation therapy is a subset of PBI that delivers a single fraction of radiation at the time of lumpectomy, saving patient and facility time and resources. There is a potential therapeutic advantage to delivering radiation to the operative tumor bed, with a steep dose fall-off and minimal dose delivered to non-involved tissues. Several IORT delivery systems are available, most of which use kilovoltage (kV) photons or megavoltage electrons.

**Figure 1:**
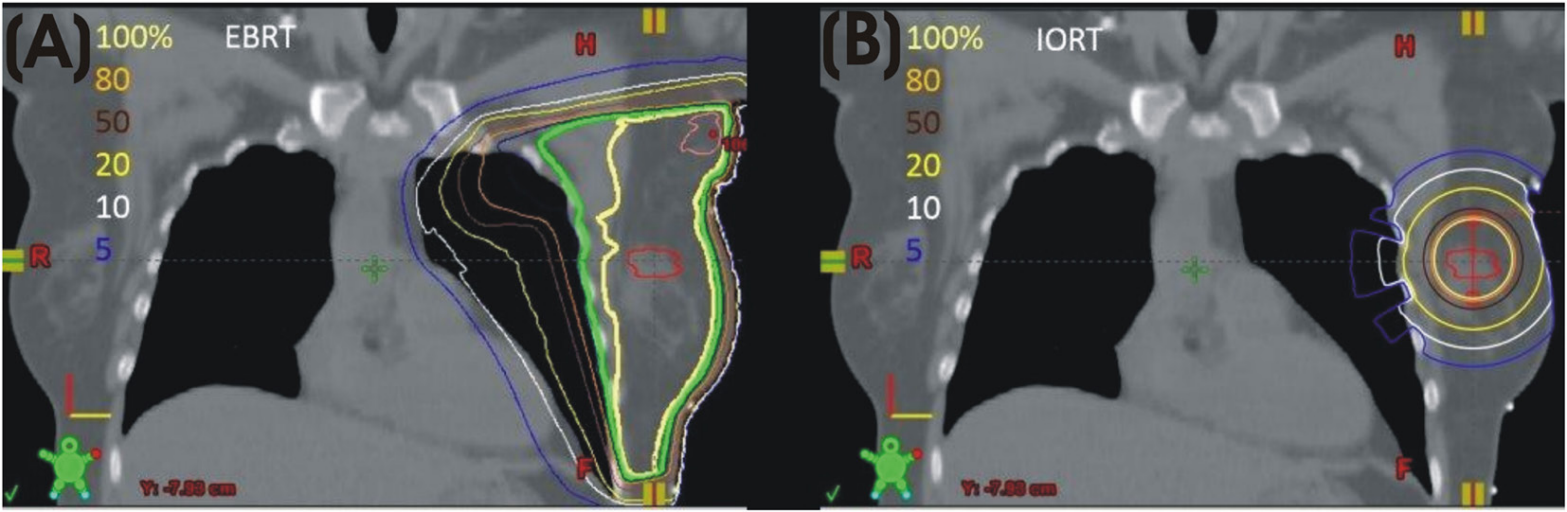
**(A)** Left breast plan with isodose lines. **(B)** Model of calculated isodose plan with 4 cm 50 kV applicator in lumpectomy site.

The intrabeam IORT device provides a point source of 50 kV X-rays at the center of a spherical applicator. Applicator diameters range from 1.5 to 5.0 cm. HVLs of the Intrabeam device correspond to effective energies of 20.7–36.3 keV. Depending on applicator diameter, the dose is reduced to 5–7 Gy at 1 cm from the applicator and 1 Gy in 2.3–6.4 cm of tissue. An *in vivo* dosimetry study showed a mean skin dose of 2.9 ± 1.6 Gy (13). A separate study demonstrated a skin dose of 0.29 ± 0.17 Gy at 5–10 cm from the applicator and a dose of 0.57 ± 0.23 Gy to the pectoral muscle in left breast patients (14). Based on these characteristics, dose to the heart and lungs from low-energy IORT is minimal. The absence of significant dose to organs at risk is a major difference between external beam radiation (Figure 1A) and low-energy, X-ray IORT (Figure 1B).

A prospective randomized trial of IORT versus whole breast RT using the Intrabeam device at the time of lumpectomy (TARGIT-A) reported a local recurrence rate at 4 years of 1.2% in the IORT group and 0.95% in the WBI group (15). Five-year results from the trial reported a 2.1% rate of breast recurrence in the IORT group versus 1.1% in the WBI group when IORT was delivered at the time of lumpectomy. When IORT was delayed until pathology was available, local recurrence was higher (5.4%) (16). Overall breast cancer mortality was the same in the TARGIT and WBI groups, but there were significantly fewer non-breast cancer deaths with TARGIT, attributable to fewer deaths from cardiovascular causes and other cancers (16).

## Lung Toxicity of WBI

Lung toxicity is a known complication of thoracic radiation that is related to dose and volume. Postmastectomy radiation in the preimage guided 3-D conformal era was associated with a significant risk of radiation pneumonitis. The use of lung constraints in treatment planning has decreased clinical lung complications associated with RT. A Swedish group showed that use of 3-D planning with a lung constraint of V20 <30% reduced the rate of radiation pneumonitis and associated decrease in pulmonary function tests (PFTs) (17). A later report from the same group showed a significant reduction in PFTs compared to pre-RT values and observed that computed tomography (CT) changes observed 4 months after RT were still detectable after 11 years (18). A prospective study of pulmonary sequelae of breast irradiation showed serial changes in lung imaging, with reduction in lung function following radiation. The study included patients receiving postmastectomy and intact breast radiation from 1996 to 1997 using 2-D planning. Lung functioning indices, including forced expiratory volume in 1 s, forced vital capacity, total lung capacity, and diffusing capacity of the lung for carbon monoxide, declined after radiation and were irreversible after 12 months (19).

Contemporary RT is CT-based and incorporates normal tissue constraints that reduce long-term toxicity. A study that coregistered quantitative single photon emission CT (SPECT)/CT of the lung with treatment plans of patients receiving breast/chest wall and regional node irradiation (V20 left lung limited to 33%) showed dose-related decreased perfusion to the lung (20). Although the use of these constraints prevented clinical symptoms of lung toxicity, patients experienced decreased lung perfusion, an indication of lung injury.

While modern radiation techniques can reduce pulmonary toxicity from external beam breast/chest wall irradiation, it occurs, is measurable, and persists long after treatment is completed.

## Cardiac Toxicity of Whole Breast Radiation

Long-term follow-up of early randomized trials for breast cancer demonstrated an increased risk of ischemic heart disease in irradiated patients, with adverse effects on survival (21). Subsequent follow-up showed that the excess of early cardiac deaths was offset by a reduction of death due to breast cancer, particularly in the more recent trials. It was postulated that earlier trials had a higher rate of cardiac toxicity because of dose and radiation techniques (2, 22).

A population-based study of breast cancer patients treated between 1958 and 2001 in Scandinavia showed that radiation to the heart during breast cancer treatment was associated with an increased risk of coronary events. The increase was proportional to mean heart dose (MHD) with no apparent threshold, started within a few years after exposure, and continued for at least 20 years. The authors estimated that a 1 Gy increase in MHD equates to a 7.4% increase in significant coronary events (23). A study of radiation dose response relationship for risk of coronary heart disease (CHD) following radiation for Hodgkin lymphoma (treated 1965–1995) showed that risk of CHD increased linearly with increasing MHD, with a median interval between treatment and expression of CHD of 19 years (24).

Both cohorts of patients were treated in the 2-D era of radiation, before widespread use of CT-based planning and adoption of current cardiac and pulmonary dose constraints. These studies demonstrate that the clinical manifestation of cardiac injury is a late effect and may not be evident until decades after treatment.

Multiple techniques have been developed to decrease cardiac dose, including prone position, intensity-modulated radiation therapy, and deep inspiration breath hold, while dose constraints have been developed to reduce late toxicity. Still, current functional studies provide evidence of measurable cardiac damage following whole breast radiation.

A prospective study of cardiac injury using resting-gated SPECT cardiac perfusion pre- and post-breast/chest wall irradiation, using CT-based planning, showed that radiation therapy caused volume-dependent perfusion defects in about 40% of patients within 6 months to 2 years of treatment. The perfusion defects were more common in patients with a larger volume of the left ventricle within the treatment field and were associated with abnormalities in regional wall motion (25).

A review of published studies of cardiac toxicity demonstrated a decrease in cardiovascular events and cardiac death rate in more modern treatment eras (26). However, the previous example illustrates that measurable cardiac injury occurs with contemporary treatment methods, though symptoms may not be immediately evident. Cardiac injury is related to dose, does not have a threshold, and may become evident years after radiation. This is a relevant concern in the treatment of breast cancer patients who are likely to survive decades after their treatment.

## Summary

Selection of radiation modality should consider treatment related-mortality and long-term pulmonary and cardiac toxicity in addition to relative risks of local recurrence and disease-free survival. Multiple techniques have been developed to decrease cardiac and lung dose, and constraints have been developed to reduce late effects. Still, current functional studies provide evidence of measurable cardiac and pulmonary damage following whole breast radiation, which should be considered when selecting treatment.

Discussion of the merits of low-energy (50 kV) IORT has often focused on the issue of local control. Less attention has been directed to potential long-term normal tissue effects. Low-energy IORT has the distinct advantage of minimal dose to the heart and lungs as well as reduced dose to uninvolved soft tissue. In selected patients, local control is not inferior to WBI. In addition, PBI, including IORT, is associated with reduced non-breast cancer mortality.

## Author Contributions

GJ: conception and design, research of supporting evidence, manuscript writing, and final approval. RS: critical revision of content, editing, creation of dosimetry model, and final approval.

## Conflict of Interest Statement

The research was conducted in the absence of any commercial or financial relationships that could be construed as a potential conflict of interest.
